# Psychometric properties of a brief, self-report measure of social inclusion: the F-SIM16

**DOI:** 10.1017/S2045796021000755

**Published:** 2022-01-21

**Authors:** Kate Filia, Caroline X. Gao, Henry J. Jackson, Jana Menssink, Amity Watson, Andrew Gardner, Sue M. Cotton, Eóin Killackey

**Affiliations:** 1 Orygen, Parkville, VIC, Australia; 2 Centre for Youth Mental Health, University of Melbourne, Parkville, VIC, Australia; 3 Department of Epidemiology and Preventative Medicine, School of Public Health and Preventive Medicine, Monash University, Melbourne, VIC, Australia; 4 Melbourne School of Psychological Sciences, University of Melbourne, Parkville, VIC, Australia; 5 School of Psychological Sciences, Monash University, Clayton, VIC, Australia

**Keywords:** Social inclusion, social exclusion, psychometrics, self report, surveys and questionnaires

## Abstract

**Aims:**

A disproportionate number of people with mental ill-health experience social exclusion. Appropriate measurement tools are required to progress opportunities to improve social inclusion. We have developed a novel measure, the Filia Social Inclusion Measure (F-SIM). Here we aimed to present a more concise, easy-to-use form, while retaining its measurement integrity by (i) refining the F-SIM using traditional and contemporary item-reduction techniques; and (ii) testing the psychometric properties of the reduced measure.

**Methods:**

Five hundred and six participants completed the F-SIM, younger and older groups of people with serious mental illness (including psychosis, mood, anxiety disorders) and same-aged community counterparts. The F-SIM was completed at baseline and 2-week follow-up, alongside other measures (including social inclusion, loneliness). The F-SIM was refined using multidimensional scaling network analysis, confirmatory factor analysis and item response theory. The psychometric evaluation included assessment of dimensionality, internal consistency, test–retest reliability, discriminant ability and construct validity.

**Results:**

The F-SIM was reduced from 135-items to 16; with 4-items in each domain of housing and neighbourhood, finances, employment and education and social participation and relationships. Psychometric properties were sound, including strong internal consistency within domains (all *α* > 0.85) and excellent overall (*α* = 0.92). Test–retest reliability was also high (*γ* = 0.90). Differences between groups were observed; clinical subgroups consistently reported lower levels of social inclusion compared to community counterparts.

**Conclusions:**

The F-SIM16 is a sound, reliable, brief self-report measure of social inclusion suitable for use in clinical and research settings. It has the potential to evaluate the effectiveness of interventions, and aid in fostering targeted and personalised needs-based care.

## Introduction

While traditionally mental health care focused on the reduction and/or elimination of distressing symptomatology (Slade *et al*., 2014; van Os *et al*., 2019), more recently the recovery movement has shifted focus to person-centred holistic care, acknowledging the importance of participation in society (Davidson, 2016). As such, in conjunction with the treatment of symptoms (Le Boutillier *et al*., 2011; Slade *et al*., 2014), there has been more consideration of education and employment, finances, housing, physical health, community participation and quality of life (van Os *et al*., 2019); areas highly valued by people with mental ill-health and their families (Connell *et al*., 2014; Robotham *et al*., 2016). Many of these domains are characteristic of social inclusion (Filia *et al*., 2018), a relatively new concept in the mental health field, with a focus on the degree to which a person participates in their communities (Productivity Commission, 2019). Due to the inter-connected nature of these domains, difficulties in one domain can impact other domains and reduce overall social inclusion. In addition, a negative cycle can ensue between social exclusion and poor mental health that, once started, can be difficult to break (Filia *et al*., 2021). However, relationships between these domains, and the impact and direction of the relationship with mental health remain underexplored.

Research on social inclusion in those impacted by mental ill-health has been hampered by a lack of definitional consensus, understanding of its components, and how to best measure the construct (Morgan *et al*., 2007; Huxley *et al*., 2012; Filia *et al*., 2018). While a number of measures of social inclusion are available (Stickley and Shaw, 2006; Lloyd *et al*., 2008; Dorer *et al*., 2009; Secker *et al*., 2009; Marino-Francis and Worrall-Davies, 2010; Huxley *et al*., 2012; Mezey *et al*., 2013), they have yet to undergo either a complete psychometric evaluation, and/or have restricted use for people with mental ill-health (Coombs *et al*., 2013; Cordier *et al*., 2017; O'Donnell *et al*., 2018). Our team has been working to address these issues, developing the Filia Social Inclusion Measure (F-SIM), with items based on a thematic analysis of the literature (Filia *et al*., 2018) and a consensus study of those impacted by mental ill-health (Filia *et al*., 2019*a*). In its original long-form, the F-SIM has demonstrated validity in discriminating the impacts of mental ill-health on social inclusion of consumers and caregivers (Filia *et al*., 2019*b*), and the preliminary characteristics of the measure have been tested in clinical and community-based youth samples (Gardner *et al*., 2019; Gardner *et al*., 2020). Refinement of this measure was required to ensure brevity and practicality of use in clinical and research settings. Thus, the aims of this work were to (i) use item reduction techniques to produce a more concise version of the measure; and (ii) test the psychometric properties of the reduced measure, including dimensionality, internal consistency, test–retest reliability, the ability to discriminate between groups, and construct validity.

## Methods

**Fig. 1.**
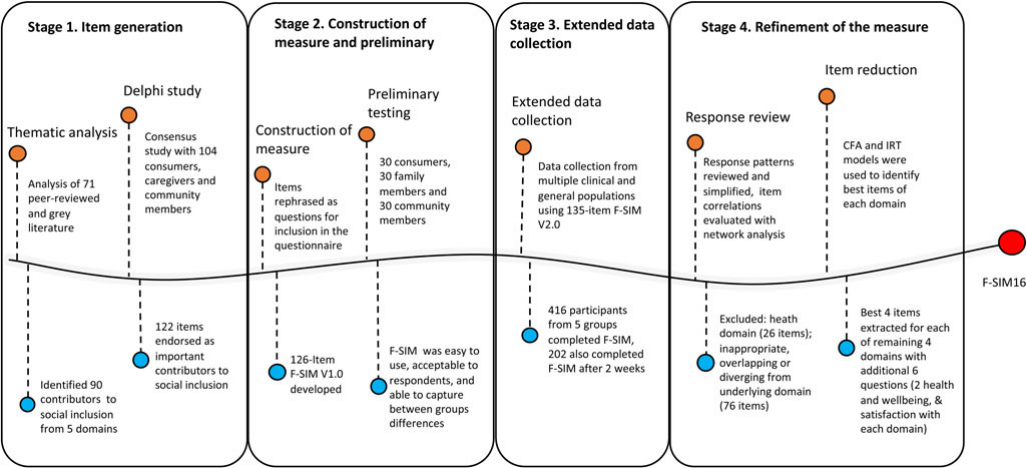
Process of Development of F-SIM16 from Stage 1 (Item Generation) to Stage 4 (Refinement and Validation). *Note:* The first iteration of the measure developed (F-SIM V1.0) in Stage 2 comprised 126 items, with a reference period of the past month. Most items were measured on a dichotomous scale (*Yes*/*No*), or Likert scales. Nine additional items were included in (F-SIM V2.0) at Stage 3 for clarity. Data collected from Stage 2 and 3 were harmonised and pooled together in Stage 4 for further psychometric evaluation.

The F-SIM was developed iteratively over several stages briefly outlined here (see Fig. 1 and for more details, Filia *et al*., 2019*b*; Gardner *et al*., 2019).

### Preliminary work

#### Stage 1. Item generation

An evidence-based definition of social inclusion was established over two studies. First, a thematic analysis of academic peer-reviewed and grey literature to identify conceptualisations of contributors of social inclusion (Filia *et al*., 2018). Second, a consensus study regarding the relative importance of contributors to social inclusion to those with a lived experience of mental ill-health, and the general community (Filia *et al*., 2019*a*).

#### Stage 2. Construction of measure and preliminary testing

Contributors identified in the thematic analysis, and agreed upon as important in the consensus study, were rephrased into question form to allow self-report administration. We grouped contributors according to the similarity of features into five overarching domains: (i) Housing and neighbourhood (HN); (ii) Social relationships, Participation and limitations (SOC); (iii) Employment and education (EE); (iv) Finances (FIN) and; (v) Health and wellbeing (HW). Preliminary psychometric evaluation of the face and discriminant validity of the 126-item version (F-SIM V1.0) in 90 participants suggested it was easy to use, highly acceptable to respondents, and able to discriminate between groups in terms of social inclusion (Filia *et al*., 2019*b*).

#### Stage 3. Extended data collection

To conduct further validation, we collected data from participants in five groups (*N* = 416): (i) young people (18–25 years) with a serious mental illness (SMI; psychosis); (ii) same-aged young people with an SMI (not psychosis); (iii) same-aged young people from the general community; (iv) older people (26 years+) with an SMI; and (v) same-aged older people from the general community. The methodology has been detailed previously (Gardner *et al*., 2019). In brief, participants completed the F-SIM Version 2.0 (a 135-item version, additional questions included for clarity), alongside other measures including an existing, albeit brief social inclusion measure (the Social Inclusion Scale, SIS; Secker *et al*., 2009), and the UCLA Loneliness Scale (UCLA-LS; Russell, 1996). A subset of participants (*n* = 202) completed the F-SIM again at a 2-week follow-up.

### Current study

#### Stage 4. Refinement of the measure

##### Participants and data

Data from Stages 2 (*N* *=* *90*) and 3 (*N* = 416) were pooled and analysed in Stage 4 (*N* = 506). Relevant measures analysed here included the F-SIM, the UCLA-LS and the SIS. Higher scores on the UCLA-LS and SIS indicate greater subjective loneliness and isolation, and degrees of social inclusion respectively.

##### Item evaluation and reduction

All analyses were conducted using R version 4.0.2 (2020-06-22). Items were refined and recoded according to their distributions (e.g. item response categories with low prevalence were combined). Items were excluded if they had a high degree of overlap (almost identical response patterns), were superseded by other broad or essential questions, or were age-specific.

In the second step, network psychometric analyses were used to understand the interplay between items, and their underlying domains (Borsboom and Cramer, 2013). We used multidimensional scaling (MDS) network analysis (Jones *et al*., 2018) of the pairwise tetrachoric correlation coefficients, *γ*_tc_, with the benefit that the distance between any pair of nodes (variables) is directly interpretable as the strength of association. MDS network analysis is additionally helpful for understanding complex associations in high-dimensional data, where other techniques such as factor analysis, can be difficult to interpret (Borsboom and Cramer, 2013; Jones *et al*., 2018). Using MDS networks we identified: (i) whether items reflected their latent domain (distance of the location from other items in the same domain); and (ii) items with a higher level of overlap.

Third, single factor confirmatory factor analysis (CFA) and Item response theory (IRT) 2PL models were used to identify the best items within each domain for inclusion in the final measure. Items selected included those more reflective of the latent domain(s) of interest (according to CFA and IRT results), and of more relevance to the theoretical framework.

##### Psychometric evaluation of the revised measure

Pairwise associations between items within each domain were evaluated using tetrachoric correlation coefficients, *r*_tc_. Item-to-total correlations within each domain were estimated using biserial correlation coefficients, *r*_bs_. Correlations were estimated using pairwise complete observations. Internal reliability was evaluated using Cronbach's alpha, *α*, based on *r*_tc_. MDS network modelling using *γ*_tc_was then carried out to obtain an overview of all items. Three CFA models were used to evaluate whether the measure was unidimensional, multidimensional, or best represented by a second-order latent factor. Overall model fit was examined using chi-square (*χ*^2^) goodness-of-fit statistics, root mean square error of approximation (RMSEA: <0.08 acceptable; 0.05 excellent), Tucker–Lewis index (TLI: >0.90 acceptable; >0.95 excellent) and comparative fit index (CFI: >0.90 acceptable; >0.95 excellent) (Hooper *et al*., 2008).

We then examined the capacity of the revised measure to distinguish between groups. First, we visually examined the distributions of domain scores (0–100) across the five groups. Second, we conducted a range of comparisons both across and within clinical and community groups using independent samples *t*-tests.

Test-retest reliability was evaluated using the 2-week follow-up data. Pearson product-moment correlations (*r*) were (i) evaluated between baseline and follow-up total scores; and (ii) used to compare the reduced version of the F-SIM with the UCLA-LS and the SIS.

## Results

### Sample characteristics

The cohort comprised 506 participants from the five different groups : (i) young people (18–25 years) with a SMI (psychosis; *n* = 149), (ii) young people (18–25 years) with a SMI (not psychosis; *n* = 26), (iii) young people (aged 18–25 years) from the general community (*n* = 163), (iv) older people (26 years+) with a SMI (*n* = 64); and (v) older people (26 years+) from the general community (*n* = 104; see Table 1).

**Table 1.** Demographic characteristics of the total cohort and five population groups

	Overall (*N* = 506)	Young				Older		
		Psychosis (*N* = 149)	Clinical not psychosis (*N* = 26)	General community (*N* = 163)	*p*-Value^a^	Clinical (*N* = 64)	General community (*N* = 104)	*p*-Value^a^
Gender					0.9			0.4
Female	272 (54%)	70 (47%)	11 (42%)	86 (53%)		37 (58%)	68 (65%)	
Male	216 (43%)	67 (45%)	12 (46%)	76 (47%)		26 (41%)	35 (34%)	
Non-binary	18 (4%)	12 (8%)	3 (12%)	1 (0.6%)		1 (2%)	1 (1%)	
Age, mean (s.d.)	27 (11)	21 (2)	22 (2)	21 (2)	0.11	41 (9)	39 (10)	0.2
Participation in the follow-up survey	202 (40%)	36 (24%)	10 (38%)	123 (75%)	<0.001	22 (34%)	11 (11%)	<0.001

a Pearson's Chi-squared for a categorical variable (excluding the non-binary group due to small cell count); Independent sample *t*-test or ANOVA test for continuous variable.

### Item evaluation and reduction

#### Item refinement

Distributions of Likert-scaled items (excluding satisfaction items) were either very skewed or bimodal, suggesting the underlying latent factor underpinning these questions was more likely categorical than continuous, or that participants were likely to respond to questions with a binary mindset. Hence, responses were converted to a binary system (*Yes*/*No*) allowing ease of comparison with other items, and simplifying response selection for respondents. Examples of binary conversions include Likert responses such as ‘1-Not at all’, ‘2-A little bit’, ‘3-Very much so’ being converted to 1 = No, 2&3 = Yes; or ‘1-Definitely limited’, ‘2-Limited a bit’, ‘3-Not at all’ being converted to 1&2 = Yes, 3 = No.

Forty-five items were excluded after item responses were evaluated for all questions, leaving 90-items remaining.

#### Item domain mapping

Fifty-seven items were excluded in this stage. Items were considered within the five domains they were initially grouped. Associations between items were mapped using MDS network analysis and data-supported four of the five domains. The remaining domain (Health and Wellbeing), was excluded from the revised measure in its entirety (26 items). MDS analysis indicated that these items did not correlate closely with one another as a cluster. Further, it was determined that health items should be measured independently of social inclusion to account for different health conditions, and allow for a clearer assessment of the relationship between both.

Twenty-five items diverging from their associated latent domain were excluded. For example, the item ‘Of the household members over the age of 18, are all currently employed or attending formal education?’ was found unrelated to the underlying HN domain. Finally, six items had a high degree of overlap with core items of the individual domain and were thus excluded. Thirty-three items remained at this point.

#### Further item reduction

CFA and IRT models of individual domains supported within-domain unidimensionality for the remaining 33 items. Four items from each domain were selected on the basis of higher factor loadings and lower residuals in the CFA model, and higher discriminant ability and ability to maximise item information in the IRT model. Where items had similar psychometric properties, those that better reflected the theoretical framework of social inclusion were chosen.

#### F-SIM16 measure

The final version of the measure comprised 16 core items, herein referred to as the F-SIM16. Table 2 contains an overview of items and associated short labels, with the full measure available upon request. An additional six items (not included in final scoring) were included as supplementary questions: two questions related to health and wellbeing (*Do you feel your emotional/physical health interferes in your ability to achieve all you would like each day?*), and four items related to satisfaction (not collected in the current study) with each of the domains. The supplementary questions provide an overview of the impact of health issues on functioning and satisfaction with domains of social inclusion, and will be validated in future studies.

**Table 2.** Questions from the 16 item version of the F-SIM

Short title	ID	Question content
Housing and neighbourhood (HN)		
Stable housing	Q1	Stability of housing
House lacking	Q2	Housing lacking, making it difficult to live in
Location not liked	Q3	Living in a less than ideal location
Neighbourhood lacking	Q4	Neighbourhood lacking in some way
Social relationships, participation and limitations (SOC)		
Good friends	Q5	Good friends to share time, experiences, thoughts and feelings with
Don't enjoy	Q6	Limited in participating socially due to a lack of enjoyment of social activities
Not participated long	Q7	Limited in participating socially due to recent lack of participation in any social or community activities
Stigma	Q8	Limited in participating socially due to regularly experiencing stigma and/or discrimination
Employment and education (EE)		
Work/study	Q9	Employment or education status
Good conditions	Q10	Work/study under good conditions
Discrimination	Q11	Limited ability to obtain or keep a job due to health-related discrimination
Impaired ability	Q12	Impaired ability to perform your occupational role or disrupt employment
Finances (FIN)		
Cover basic costs	Q13	Enough income to cover basic everyday costs
Unable to participate	Q14	Inability to participate in social activities due to income
Unable to attend events	Q15	Inability to attend important events such as weddings, funerals, birthday celebrations due to income
Lack savings	Q16	Lack of savings for use in an emergency
Supplementary health and wellbeing (HW)		
Limited by physical health	Q1+	Ongoing physical ailments that prevent you from achieving all you would like in your life
Limited by emotional health	Q2+	Ongoing emotional health concerns that interfere in your ability to achieve all you would like in your life

### Psychometric evaluation of the F-SIM16

#### Inter-item correlations

Pairwise *r*_tc_ between individual items within domains, item-to-total biserial correlations (*r*_bs_) and Cronbach's alpha within each domain are provided in Table 3. Inter-item correlations within each domain ranged from medium to strong. Item-to-total biserial correlations (*r*_bs_) ranged between 0.75 (HN and *Stable housing*) and 0.89 (FIN and *Unable participate*). Each domain showed strong internal consistency (*α* > 0.85) and the internal consistency for the 16 items was excellent (*α* = 0.92). Individual item correlations (provided in online Fig. S1 in Supplementary Material) were visualised using network analysis (see online Supplementary Fig. S2). Most items were closely related to items within their overarching domains. The *Discriminatio*n and *Impaired ability* items in the EE domain had moderate correlations with items in the SOC domain.

**Table 3.** Inter-item tetrachoric correlations (*r*_tc_), item-to-total polyserial correlations (*r*_bs_) and Cronbach's alphas (*α*) for each social inclusion domain

						*α*
Housing and neighbourhood (HN)	*r*_tc_	*r*_tc_	*r*_tc_	*r*_tc_	*r*_ps_	0.929
	(1)	(2)	(3)	(4)	HN total score	
Stable housing (1)	1.000				0.747	
House lacking^a^ (2)	0.896	1.000			0.795	
Location not like^a^ (3)	0.833	0.693	1.000		0.883	
Neighborhood lacking^a^ (4)	0.877	0.570	0.722	1.000	0.821	
Social participation, relationships and limitations (SOC)						0.854
	(5)	(6)	(7)	(8)	SOC total score	
Good friends (5)	1.000				0.814	
Don't enjoy^a^ (6)	0.598	1.000			0.854	
Not participated long^a^ (7)	0.671	0.705	1.000		0.877	
Stigma^a^ (8)	0.399	0.572	0.615	1.000	0.788	
Employment and education (EE)						0.877
	(9)	(10)	(11)	(12)	EE total score	
Work/study (9)	1.000				0.810	
Good conditions (10)	0.999^b^	1.000			0.861	
Discrimination^a^ (11)	0.449	0.589	1.000		0.835	
Impaired ability^a^ (12)	0.427	0.530	0.853	1.000	0.854	
Finances (FIN)						0.918
	(13)	(14)	(15)	(16)	FIN total score	
Cover basic costs (13)	1.000				0.797	
Unable participate^a^ (14)	0.660	1.000			0.888	
Unable attend events^a^ (15)	0.620	0.857	1.000		0.871	
Lack savings^a^ (16)	0.709	0.813	0.767	1.000	0.883	

*r*_pc_, tetrachoric correlation coefficient; *r*_ps_, polyserial correlation coefficient; *α*, Cronbach's alpha.

a Reverse scored.

b High correlation here is related to these being nested questions, a high proportion of participants working suggested that they were working in a good condition.

#### Overall instrument evaluation

**Fig. 2.**
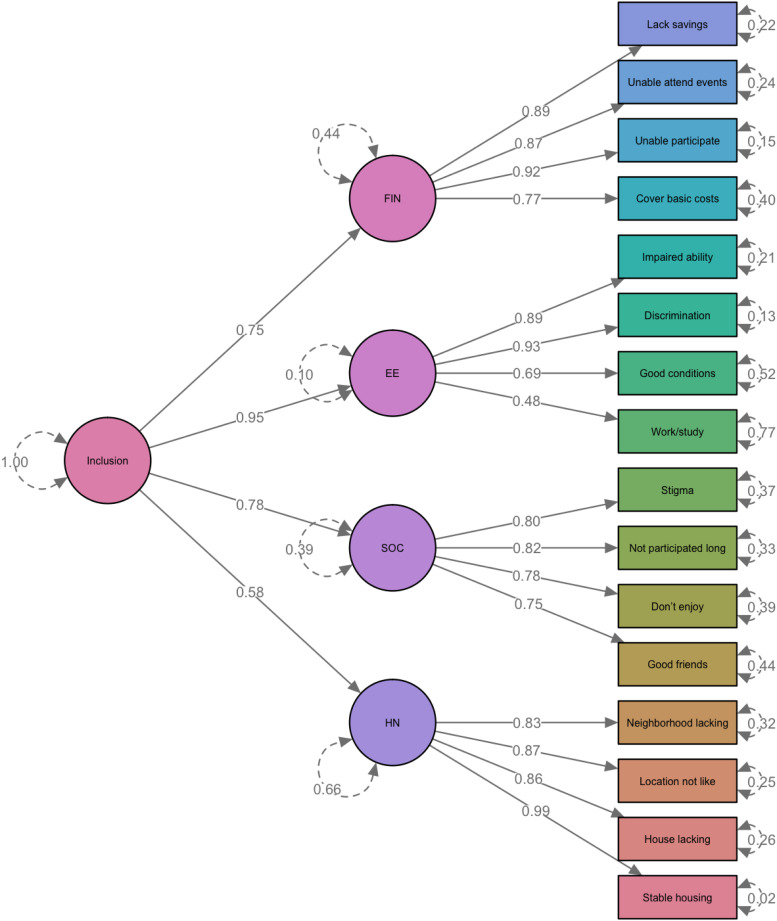
Factor Loading and Residual Variance Estimated from the Second-order CFA Model. *Reverse scored items. CFA model was based on 488 records with complete data.

Model fit indicators for the three CFA models are provided in online Supplementary Table S1. Both the four-factor and second-order models had an excellent fit with the data (RMSEA < 0.05; CFI/TLI > 0.95). With fewer parameters, the second-order model was preferred. Factor loadings and residual variances estimated for the second-order CFA are displayed in Fig. 2. The second-order model supports the calculation of domain scores, in addition to the total score.

#### Group differences

Distributions of the F-SIM16 domain and total scores, as well as individual item responses by groups, are provided in online Supplementary Table S2 and Fig. S3. Young people with psychosis or other mental ill-health reported the lowest level of social inclusion in all domains, particularly in the SOC and FIN domains. Although a proportion of older participants from the clinical population reported a lower level of social inclusion, the subgroup experienced a similar level of inclusiveness compared with the general community (reflected by the bimodal distribution in SOC and FIN domain). Pairwise comparisons of the domain and total scores, as shown in online Supplementary Table S3, suggested that clinical subgroups consistently reported lower levels of social inclusion compared to community counterparts across all domains. Participants from the general community groups were more comparable in all domains irrespective of age, except for lower scores in the SOC domain among young people.

#### Test-retest reliability

The correlation coefficient (*r*) between baseline and follow-up scores of individual domains varied between 0.68 (HN) and 0.83 (EE and FIN; SOC 0.73). The F-SIM16 total score had a correlation (*γ*) of 0.90 between baseline and follow-up, indicative of high test-retest reliability.

#### Correlation with similar constructs

Both the SIS and UCLA Loneliness Scale had strong correlations with the F-SIM16 total score (*r* *>* 0.60) (online Supplementary Table S4). As expected, the SIS and UCLA-LS had low correlations with some of the F-SIM16 domains including FIN, EE and HN.

## Discussion

Social inclusion is an important concept in mental health, particularly with an increasing focus on consumer-driven outcomes and recovery. To facilitate continued and meaningful progress, novel outcome measures are required. Here we reported on a novel measure of social inclusion, the F-SIM, detailing refinement to a 16-item form (the F-SIM16), using traditional and contemporary psychometric techniques.

### The F-SIM16

The F-SIM16 is a brief, easy to use, self-report measure with sound psychometric properties validated in populations of younger and older people with mental ill-health and same-aged community peers. Items were selected using a combination of data and theory-driven approaches. As intentionally designed, items remain a combination of subjective and objective characteristics of social inclusion within each domain.

Psychometric analyses supported the four core domains of social inclusion, as identified in our earlier work (Filia *et al*., 2018) and endorsed by considerable stakeholder engagement (Filia *et al*., 2019*a*, 2019*b*). We observed high-level internal consistency within domains and an excellent second-order factor model fit, which supported the theoretical underpinnings of this multifarious concept. The cohesive network association also demonstrated multidimensionality, and highlighted interconnected elements.

The measure was found to have good discriminant ability, demonstrating differences in the domain and total scores across different populations, potentially reflective of the particular challenges these groups face as a result of age and/or experience of mental ill-health. This was particularly evident in findings that clinical groups (irrespective of age) reported poorer social inclusion than same-aged general community peers. Young clinical groups also reported lower levels of social inclusion overall compared with the older clinical group. This could indicate that over time older people with mental ill-health find ways of adapting to their circumstances, perhaps having secured stable accommodation, receiving and finding ways to manage finances, and connecting socially in ways that are meaningful to them.

The F-SIM16 demonstrated temporal robustness, making it an excellent tool for evaluating the impact of interventions.

### Other measures of social inclusion

As the F-SIM16 was developed in response to an identified need for appropriate measures of social inclusion, so too were other measures, including the SIS (Stickley and Shaw, 2006; Lloyd *et al*., 2008; Dorer *et al*., 2009; Secker *et al*., 2009; Marino-Francis and Worrall-Davies, 2010; Huxley *et al*., 2012; Mezey *et al*., 2013). As per the F-SIM16, the development and evaluation of some of these measures have progressed (Huxley *et al*., 2015; Wilson and Secker, 2015; Mezey *et al*., 2020), yet none have reported a complete psychometric evaluation to date. Additional limitations include poor generalisability as a result of having been developed for use in specific settings (Stickley and Shaw, 2006; Dorer *et al*., 2009; Secker *et al*., 2009; Marino-Francis and Worrall-Davies, 2010) and imbalances in the inclusion of objective/subjective elements (either a greater focus on subjective measures [Stickley and Shaw, 2006; Lloyd *et al*., 2008; Secker *et al*., 2009; Marino-Francis and Worrall-Davies, 2010), or an entire focus on objective measures (Dorer *et al*., 2009)]. Stakeholder engagement also varied considerably, from comprehensive consultation on all stages of measure development (Stickley and Shaw, 2006), to engagement post-measure construction (Marino-Francis and Worrall-Davies, 2010; Mezey *et al*., 2013). Comparatively, the F-SIM16 possesses a solid theoretical foundation, significant stakeholder engagement during development and testing, generalisability across different groups, and a balance of subjective and objective components of social inclusion across multiple domains. Comparing the F-SIM to other measures indicated that it was the superior tool for measuring social inclusion as a multidimensional construct, with the UCLA-LS and SIS both appearing to measure one domain more generally (SOC).

### Limitations

The main limitation of the current research relates to the psychometric evaluation utilising data collected from the original version of the F-SIM. Future studies are needed to further evaluate the psychometric properties of the F-SIM16, and assess the utility of the additional variables (health and wellbeing, and satisfaction with domains). A further limitation is that test-retest validity included a small sample of participants, in part due to low retention of participants. As a result, longitudinal patterns and responses to change are largely unknown. Additional research to address the above is needed.

### Strengths and implications

Refining the F-SIM to the F-SIM16, provides a very useful tool with broad clinical and research utility. The F-SIM16 has the potential to contribute significantly to the broader understanding of social inclusion in mental ill-health, and to increase understanding of the dynamics between mental health and social inclusion. The brief, self-report nature enables the collection of wide-scale data via online approaches, ensuring accessibility and completion of the survey by those potentially most excluded, thus reducing bias in assessments, and at a low-cost.

The properties of the F-SIM16 enable the collection of sound empirical data, including data for at-risk, consumer groups and normative data. This implication is by far the most valuable, providing information on the distribution of scores across population groups, and identifying strengths and areas impacted in specific domains. Further, the inclusion of the additional questions related to individual's satisfaction with each domain will ensure we maintain an understanding of how personally satisfied individuals are with their unique circumstances.

The F-SIM16 will aid in determining the effectiveness of programs and interventions designed to improve social inclusion. It will allow for pre- and post-measurement, and the identification of those aspects of social inclusion most receptive to change. This will foster the development of more informed and relevant interventions. The ability to provide data about the extent of social inclusion in various populations (geographical, diagnostic, with respect to age, gender etc.) can inform policy development and service reform to improve both social inclusion, and the health and wellbeing of people with mental illness.

From a clinical perspective, this tool will assist in tailoring treatment plans, and determining the need for collaborative approaches to treatment. Completing a measure of social inclusion at the outset of treatment allows for the charting of progress across time and facilitates more holistic outcome measurement. Completion at routine time points, as well as at times of illness exacerbation, may assist in determining individual risk factors and the relationship between symptomatology and social inclusion, to aid in treatment and maintaining wellbeing.

Together these strengths demonstrate the significant contribution of the F-SIM16 to the field of mental health, with the potential for considerable impact by providing a more rigorously developed, comprehensive measure of social inclusion than previously available. Implementation of the F-SIM16 in research or clinical evaluations to determine treatment, service gaps and needs, and to target facilitators of social inclusion, has significant potential to improve the lives of people with SMI.

## Data Availability

The data that support the findings of this study are available upon reasonable request from the corresponding author (KF) and subject to ethical approval/restrictions.

## Supplementary material

For supplementary material accompanying this paper visit https://doi.org/10.1017/S2045796021000755. click here to view supplementary material
