# Supplementary material for: Psychometric properties of a brief, self-report measure of social inclusion: the F-SIM16
Source: Epidemiol Psychiatr Sci. 2022 Jan 21;31:e8. doi: 10.1017/S2045796021000755 (PMC8786614; doi:10.1017/S2045796021000755)
Supplement: Supplementary file 1 [file S2045796021000755sup001.docx]

**Psychometric properties of a brief, self-report measure of social inclusion - the F-SIM16**

**(Supplementary material)**

**Figure S1**

*Tetrachoric Correlation Coefficients (Associated P-value) Between the Individual Items of the F-SIM16*


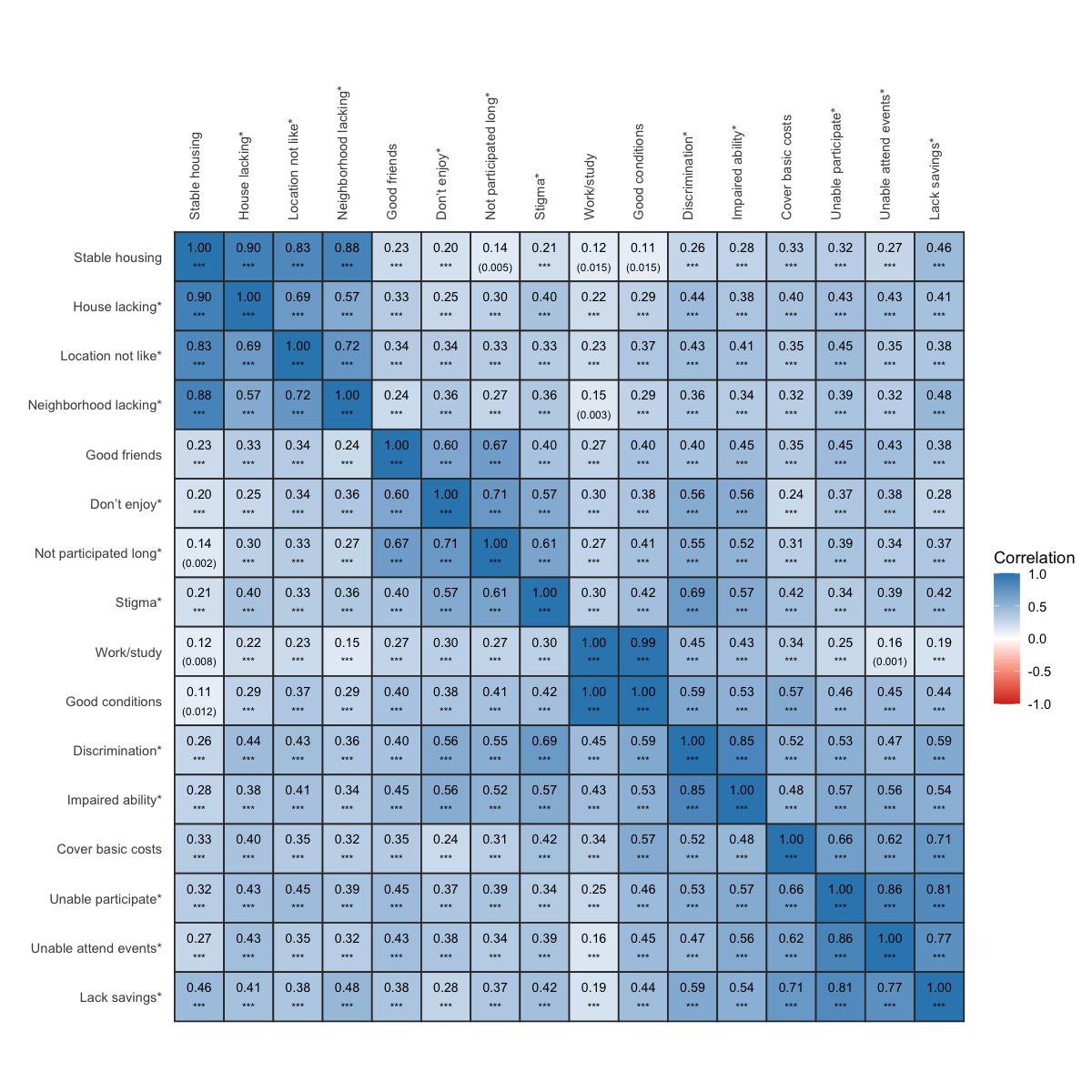


*. * Reverse scored items; *** p-value<0.001*

**Figure S2**

*MDS Network Plot of Tetrachoric Correlation Coefficients Between the Individual Items of the F-SIM16*


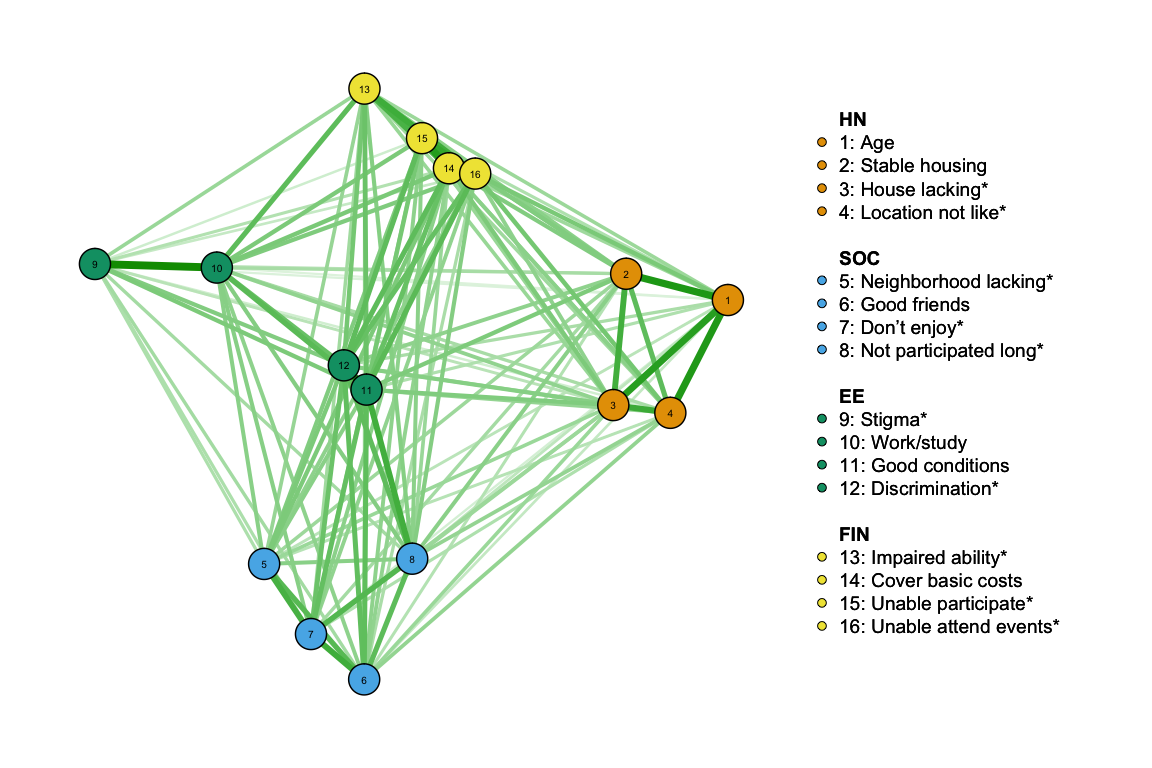


** Reverse scored items*

**Table S1**

*Fitting Indices of Three CFA models based on 488 records with complete data*

| **Model** | **Chi2** | **df** | **RMSEA** | **CFI** | **TLI** |
| --- | --- | --- | --- | --- | --- |
| One-factor CFA | 640 | 104 | 0.103 | 0.93 | 0.92 |
| Four-factor CFA | 90 | 98 | 0.001 | 1.00 | 1.00 |
| Second-order CFA | 105 | 100 | 0.010 | 1.00 | 1.00 |

**Table S2**

*Responses for the Total Cohort and 5 Population Groups for F-SIM16 Items, Total Score and Sub-domain Total Scores*

|  | **Overall**  **(N = 506)** | **Young Psychosis**  **(N = 149)** | **Young Clinical Not Psychosis**  **(N = 26)** | **Young General Community**  **(N = 163)** | **Older Clinical  (N = 64)** | **Older General Community**  **(N = 104 )** |
| --- | --- | --- | --- | --- | --- | --- |
| **Housing and Neighbourhood (HN)** | | | | | | |
| Stable housing | 489 (97%) | 135 (91%) | 26 (100%) | 160 (98%) | 64 (100%) | 104 (100%) |
| House hacking | 73 (15%) | 35 (24%) | 7 (28%) | 12 (7.4%) | 10 (16%) | 9 (8.7%) |
| Location not like | 115 (23%) | 53 (36%) | 6 (24%) | 25 (15%) | 21 (33%) | 10 (9.6%) |
| Neighborhood lacking | 85 (17%) | 46 (31%) | 5 (20%) | 16 (9.8%) | 11 (17%) | 7 (6.8%) |
| **HN total score** | 86 (25) | 75 (32) | 82 (23) | 91 (20) | 84 (22) | 94 (15) |
| **Social Relationships, Participation and Limitations (SOC)** | | | | | | |
| Good friends | 378 (75%) | 80 (54%) | 18 (69%) | 144 (88%) | 46 (73%) | 90 (87%) |
| Don’t enjoy | 261 (52%) | 118 (79%) | 22 (85%) | 65 (40%) | 34 (53%) | 22 (21%) |
| Not participated long | 221 (44%) | 101 (68%) | 20 (77%) | 53 (33%) | 27 (42%) | 20 (19%) |
| Stigma | 134 (27%) | 71 (48%) | 11 (42%) | 24 (15%) | 22 (34%) | 6 (5.8%) |
| **SOC total score** | 63 (34) | 40 (31) | 41 (32) | 75 (28) | 61 (35) | 85 (23) |
| **Employment and Education (EE)** | | | | | | |
| Work/study | 440 (87%) | 114 (77%) | 23 (92%) | 153 (94%) | 53 (83%) | 97 (94%) |
| Good conditions | 385 (77%) | 80 (54%) | 20 (80%) | 149 (92%) | 44 (70%) | 92 (91%) |
| Discrimination | 115 (23%) | 77 (52%) | 11 (44%) | 8 (4.9%) | 19 (31%) | 0 (0%) |
| Impaired ability | 187 (37%) | 114 (77%) | 19 (79%) | 6 (3.7%) | 40 (66%) | 8 (7.8%) |
| **EE total score** | 76 (31) | 51 (31) | 61 (28) | 94 (15) | 64 (32) | 94 (14) |
| **Finances (FIN)** |  |  |  |  |  |  |
| Cover basic costs | 363 (73%) | 74 (50%) | 16 (70%) | 136 (84%) | 44 (69%) | 93 (91%) |
| Unable participate | 199 (40%) | 98 (66%) | 14 (61%) | 36 (22%) | 28 (44%) | 23 (23%) |
| Unable attend events | 91 (18%) | 50 (34%) | 7 (30%) | 8 (4.9%) | 17 (27%) | 9 (8.8%) |
| Lack savings | 205 (41%) | 100 (67%) | 15 (65%) | 35 (22%) | 32 (50%) | 23 (23%) |
| **FIN total score** | 68 (36) | 46 (37) | 53 (36) | 84 (24) | 62 (38) | 84 (27) |
| **F-SIM16 total score** | 73 (23) | 53 (22) | 58 (19) | 86 (12) | 67 (23) | 89 (11) |
| **SUPPLEMENTARY QUESTIONS (HW) - Limited by:** | | | | | | |
| Physical health | 122 (25%) | 52 (35%) | 11 (48%) | 17 (11%) | 25 (40%) | 17 (17%) |
| Emotional health | 253 (51%) | 120 (81%) | 21 (91%) | 52 (32%) | 39 (62%) | 21 (21%) |

Note: F-SIM16 total score and subscale total scores were all scaled between 0 and 100. Statistics reported are counts and percentages for individual items and mean and SD for total scores. Missing data for individual item include: 1 for stable housing and good friends; 2 for don’t enjoy, not participated long, stigma, 3 for location not like, neighbourhood lacking, work/study; 4 for housing lacking; 6 for Good conditions, discrimination, cover basic costs, unable participate, unable attend events, lack savings, 7 for impaired ability, and 10 for limited by physical health and limited by emotional health. The missing data for total scores are 6 for HN, 3 for SOC, 9 for EE, 6 for FIN and 18 for F-SIM16 total score.

**Table S3**

*Comparisons between Sub-groups, Sub-scale and Total Scores of the F-SIM16*

|  | **Young** | | |  | **Older** | | |
| --- | --- | --- | --- | --- | --- | --- | --- |
|  | **Clinical**  **(N = 175)** | **General**  **Community**  **(N = 163)** | **p-value** |  | **Clinical  (N = 64)** | **General**  **Community**  **(N = 104)** | **p-value** |
| **NH total score** | 76 (31) | 91 (20) | <0.001 |  | 84 (22) | 94 (15) | 0.003 |
| **SOC total score** | 40 (31) | 75 (28) | <0.001 |  | 61 (35) | 85 (23) | <0.001 |
| **EE total score** | 52 (31) | 94 (15) | <0.001 |  | 64 (32) | 94 (14) | <0.001 |
| **FIN total score** | 47 (37) | 84 (24) | <0.001 |  | 62 (38) | 84 (27) | <0.001 |
| **F-SIM16 total score** | 54 (22) | 86 (12) | <0.001 |  | 67 (23) | 89 (11) | <0.001 |
|  | **Clinical** | | |  | **General community** | | |
|  | **Young**  **(N = 175)** | **Older**  **(N = 64)** | **p-value** |  | **Young  (N = 163)** | **Older**  **(N = 104)** | **p-value** |
| **NH total score** | 76 (31) | 84 (22) | 0.034 |  | 91 (20) | 94 (15) | 0.278 |
| **SOC total score** | 40 (31) | 61 (35) | <0.001 |  | 75 (28) | 85 (23) | 0.002 |
| **EE total score** | 52 (31) | 64 (32) | 0.011 |  | 94 (15) | 94 (14) | 0.940 |
| **FIN total score** | 47 (37) | 62 (38) | 0.007 |  | 84 (24) | 84 (27) | 0.872 |
| **F-SIM16 total score** | 54 (22) | 67 (23) | <0.001 |  | 86 (12) | 89 (11) | 0.036 |

Note: Statistics presented: Mean (SD), and statistical tests performed: t-test using pair-wise complete data. As the sample size of the youth clinical (not psychosis) group was much smaller compared with other groups, it was combined with the youth psychosis group for pairwise comparisons

**Figure S3**

*Domain Mean Score Distributions (Smoothed Density Plots) by Population Group*


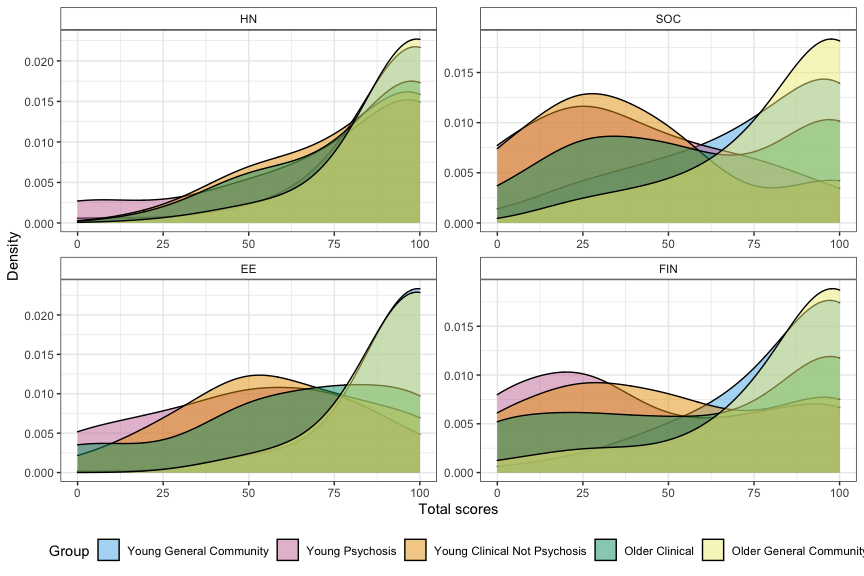


**Table S4**

*Correlations (*$r)$*with SIS, UCLA-LS*

|  | **HN** | **SOC** | **EE** | **FIN** | **F-SIM** |
| --- | --- | --- | --- | --- | --- |
| **SIS** | 0.30 | 0.73 | 0.60 | 0.51 | 0.74 |
| **UCLA Loneliness Scale** | -0.32 | -0.71 | -0.48 | -0.46 | -0.68 |

Note: p<0.001 for all $r$
